# Rubella Epidemics and Genotypic Distribution of the Rubella Virus in Shandong Province, China, in 1999–2010

**DOI:** 10.1371/journal.pone.0042013

**Published:** 2012-07-24

**Authors:** Changyin Wang, Zhen Zhu, Qing Xu, Aiqiang Xu, Xueqiang Fang, Lizhi Song, Weixiu Li, Ping Xiong, Wenbo Xu

**Affiliations:** 1 Shandong Provincial Key Laboratory for Infectious Disease Control and Prevention, Shandong Center for Disease Control and Prevention, Jingshi Road, Jinan, People’s Republic of China; 2 WHO WPRO Regional Reference Measles/Rubella laboratory, National Institute for Viral Disease Control and Prevention, Chinese Center for Disease Control and Prevention, Changbai Road, Changping District, Beijing, People’s Republic of China; University of Hong Kong, Hong Kong

## Abstract

**Background:**

The rubella vaccine was introduced into the immunization program in 1995 in the Shandong province, China. A series of different rubella vaccination strategies were implemented at different stages of measles control in Shandong province.

**Methodology/Principal Findings:**

The average reported incidence rate of rubella cases remained at a low level in Shandong province after 1999. However, rubella epidemics occurred repeatedly in 2001/2002, 2006, and 2008/2009. The age of the onset of rubella cases gradually increased during 1999–2010, which showed that most cases were found among the 10 years old in 1999 and among the 17 years old in 2010. Phylogenetic analysis was performed and a phylogenetic tree was constructed based on the World Health Organization standard sequence window for rubella virus isolates. All rubella viruses isolated in Shandong province were divided into 4 genotypes: 1E, 1F, 2A, and 2B. Genotype 1E viruses accounted for the majority (79%) of all these viruses. The similarity of nucleotide and amino acid sequences among genotype 1E viruses was 98.2–100% and 99.1–100%, respectively. All Shandong genotype 1E strains, differed from international genotype 1E strains, belonged to cluster 1 and interdigitated with the viruses from other provinces in mainland China. The effective number of infections indicated by a Bayesian skyline plot remained constant from 2001 to 2009.

**Conclusions/Significance:**

The gradual shift of disease burden to an older age group occurred after a rubella-containing vaccine was introduced into the childhood immunization schedule in 1995 in Shandong province. Four genotypes, including 1E, 1F, 2A, and 2B, were found in Shandong province during 2000–2009. Genotype 1E, rather than genotype 1F, became the predominant genotype circulating in Shandong province from 2001. All Shandong genotype 1E viruses belong to the genotype 1E/cluster 1; they have constantly circulated, and co-evolved and co-circulated, with those from other provinces.

## Introduction

The rubella virus is the etiological agent of a disease known as rubella (or German measles) [1]. The disease is generally benign, and infection is often asymptomatic. As rubella virus is a potent, infectious, and teratogenic agent, virus infection of non-immunized women during the early stages of pregnancy, particularly during the first 16 weeks, can result in miscarriage, fetal death, or an infant born with birth defects such as congenital rubella syndrome (CRS) [1], [2], [3]. In 2000, therefore, the World Health Organization (WHO) recommended the use of rubella-containing vaccine (RCV) in all countries with national childhood immunization schedules to prevent congenital rubella infection, including CRS [4]. The number of WHO Member States using RCV increased from 83 (43%) in 1996 to 130 (67%) in 2009. The number of rubella cases reported dramatically decreased from 670,894 in 2000 to 121,344 in 2009 [5].

RuV is a small, enveloped virus in the *Rubivirus* genus of the Togavirus family. The genome of RuV is a single-stranded, positive-sense RNA of approximate 10 kb. The genome encodes 2 open reading frames (ORFs), the 3′-proximal ORF (structural protein ORF) encodes the virion proteins, the nucleocapsid protein (C), and the 2 envelope glycoproteins (E2 and E1) [3]. The 739-nt region (nt 8731–9469) within the E1 glycoprotein contains important functional domains including a hemagglutination inhibiting and neutralizing epitope, and antigenic sites [6], and has been designated as WHO standard sequence window for rubella phylogeny [7]. Thus far, 2 clades including 13 genotypes have been described: clade 1 is divided into 10 genotypes (1a, 1B, 1C, 1D, 1E, 1F, 1G, 1h, 1i, and 1j); clade 2 contains 3 genotypes (2A, 2B, and 2C) [8]. Molecular epidemiology studies in China of the rubella virus indicated that 5 genotypes, including 1a, 1E, 1F, 2A, and 2B, were present in China from 1979 [9], [10]. Genotype 1E virus was the predominant virus circulating in China from 2001 through 2009 [10].

Shandong province, located in eastern China, is a developed province with a population of 94.7 million in 2009 (data from the Chinese Statistics Bureau). The province is composed of 17 prefectures, 140 counties, 1931 townships, and 88,925 administrative villages. In order to control rubella epidemics, the rubella vaccine was introduced into immunization program of Shandong province in China in 1995. From 1995 to 2007 a financial charge was made for rubella vaccination; from 2008, following the introduction of the rubella vaccine into the national Expanded Program on Immunization (EPI), all children have the opportunity to be vaccinated against rubella virus free of charge. In 1995–2007, a 2-dose schedule using monovalent rubella vaccine administered between 8–18 months of age and at 7 or 12 years old was followed, which was consistent with the measles vaccine strategy during the measles control stage. During the measles elimination stage in China, in order to eliminate measles and control rubella simultaneously, a combined measles and rubella vaccine was used; this vaccine was administered at 8 months of age in 2008 and the second dose (measles–mumps–rubella vaccine) was added at 18–24 months of age in 2010 in the western region of Shandong and extended to the whole province in 2011. Two types of rubella vaccine, including domestic vaccine (BRDII vaccine, genotype 2A) for routine immunization, and imported vaccine (RA27/3 vaccine, genotype 1a) as a selective vaccine, have been used in Shandong province since 1995. In a trial comparing the BRDII and RA27/3 vaccines, the seroconversion rates and incidence of mild side effects were found to be similar [11].

Integrated rubella surveillance including virological surveillance in Shandong province was initiated in 1999 when a measles project was conducted [12]. In this study, the epidemiological profile of rubella and the molecular epidemiology of rubella viruses isolated in Shandong province were reviewed in detail over the last 12 years when a series of different vaccination strategies were implemented with the changes in measles and rubella control policy. We sought to provide the experience of rubella surveillance to other countries.

## Results

### Survey of Rubella Cases in Shandong Province

**Figure 1:**
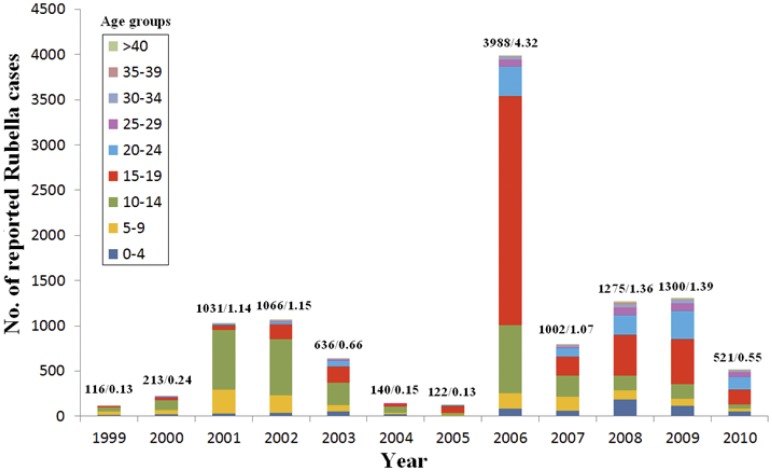
Age distribution of rubella cases in Shandong province, China, 1999–2010. The number above each column represents the number of reported rubella cases and average rubella incidence (per 100,000) in Shandong province, China.

Though the average reported incidence rate of rubella cases remained at a low level in Shandong province, rubella epidemics occurred in 2001/2002 (reported peak annual incidence rate 1.15/100,000, 1066 cases), 2006 (4.32/100,000, 3988 cases), and 2008/2009 (1.38/100,000, 1300 cases) (Figure 1). The reported rubella cases during 1999–2004 were concentrated in the group of children aged 10–14 years, with the proportion being 44.8%, 51.2%, 63.8%, 58.2%, 39.6%, and 51.8% for each year. Although the reported rubella cases within the 15–19-year-old group dramatically increased in 2005 and 2006 with the proportion being 63.9% and 63.6%, respectively, the proportion then decreased to 26.5% in 2007, and remained at approximately 35% between 2008 and 2010. The proportion of reported rubella cases within the 20–24-year-old group gradually increased from 2005 (5.7%) to 2010 (27.1%). The reported rubella cases within the 25–39-year-old group were also slightly increased. The incidence of rubella cases in children under 10 years of age was maintained at a low level between 1999 and 2010 (Figure 1). The age of the onset of rubella cases in Shandong province gradually increased during 1999–2010, which showed that most cases were found among the 10 years old in 1999 and among the 17 years old in 2010. The proportion of rubella cases in individuals aged <15 years in Shandong province was less than the central and western provinces of China, while the proportion of reported rubella cases within the 15 to 39 age group was higher than that in central and western China [10].

### Genotype Distribution of Shandong Rubella Viruses

A total of 34 rubella virus strains were isolated from throat swabs in 8 of 17 prefectures in Shandong province during 1999–2009 (Table S1). Some nucleotide sequences of the viral isolates from the outbreaks were identical in the 739-nt region within the E1 gene, and only 1 was selected for further analysis when the sequences were the same; finally, 24 rubella viruses were selected as representative viruses for phylogenetic analysis. All the viruses were named according to the WHO systematic nomenclature for rubella viruses [7].

**Figure 2:**
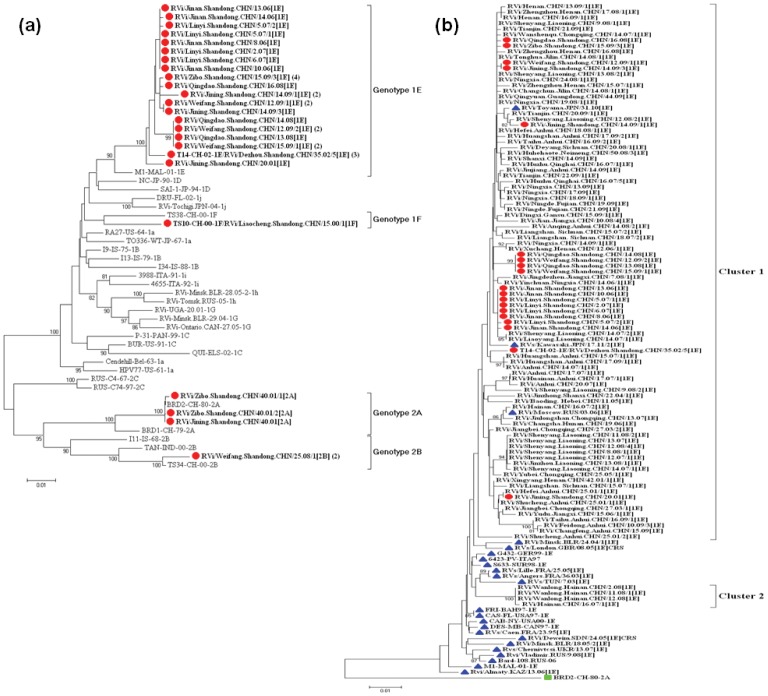
Phylogenetic analyses of sequences of Shandong rubella viruses based on the WHO standard sequence window. (a) Phylogenetic analysis of sequences of 24 representative rubella viruses from 2000–2009 compared with the WHO reference sequences. Numbers in parentheses are numbers of identical sequences found in the same outbreak. Shandong rubella viruses are indicated by a solid circle. (b) Phylogenetic analysis of representative Shandong rubella virus strains of genotype 1E compared with rubella viruses from the other provinces of China. Seventy-seven genotype 1E rubella virus strains from other provinces isolated during 2001–2009 were obtained from Genbank. Shandong rubella viruses are indicated by red solid circles, the genotype 1E rubella virus strains from other countries are indicated by blue solid triangle. The Chinese vaccine strain BRD II with a green solid square was used as an out-group.

The 24 rubella sequences were divided into 4 genotypes: genotype 1E (19 strains), genotype 1F (1 strain), genotype 2A (3 strains), and genotype 2B (1 strain). These genotype assignments were supported by high bootstrap scores except genotype 1F (Figure 2a), which was supported by the larger nucleotide difference compared with other genotypes (88.7%–94.9%). Because Shandong province firstly performed detailed molecular epidemiology study in China, comprehensive and valuable information was obtained about rubella genotype distribution. The genotypes of rubella viruses found in Shandong province covered most of the genotypes found in China, as the results of molecular epidemiology studies indicate that only 5 (1a, 1E, 1F, 2A, and 2B) genotypes have been present in China since 1979 [10].

**Figure 3:**
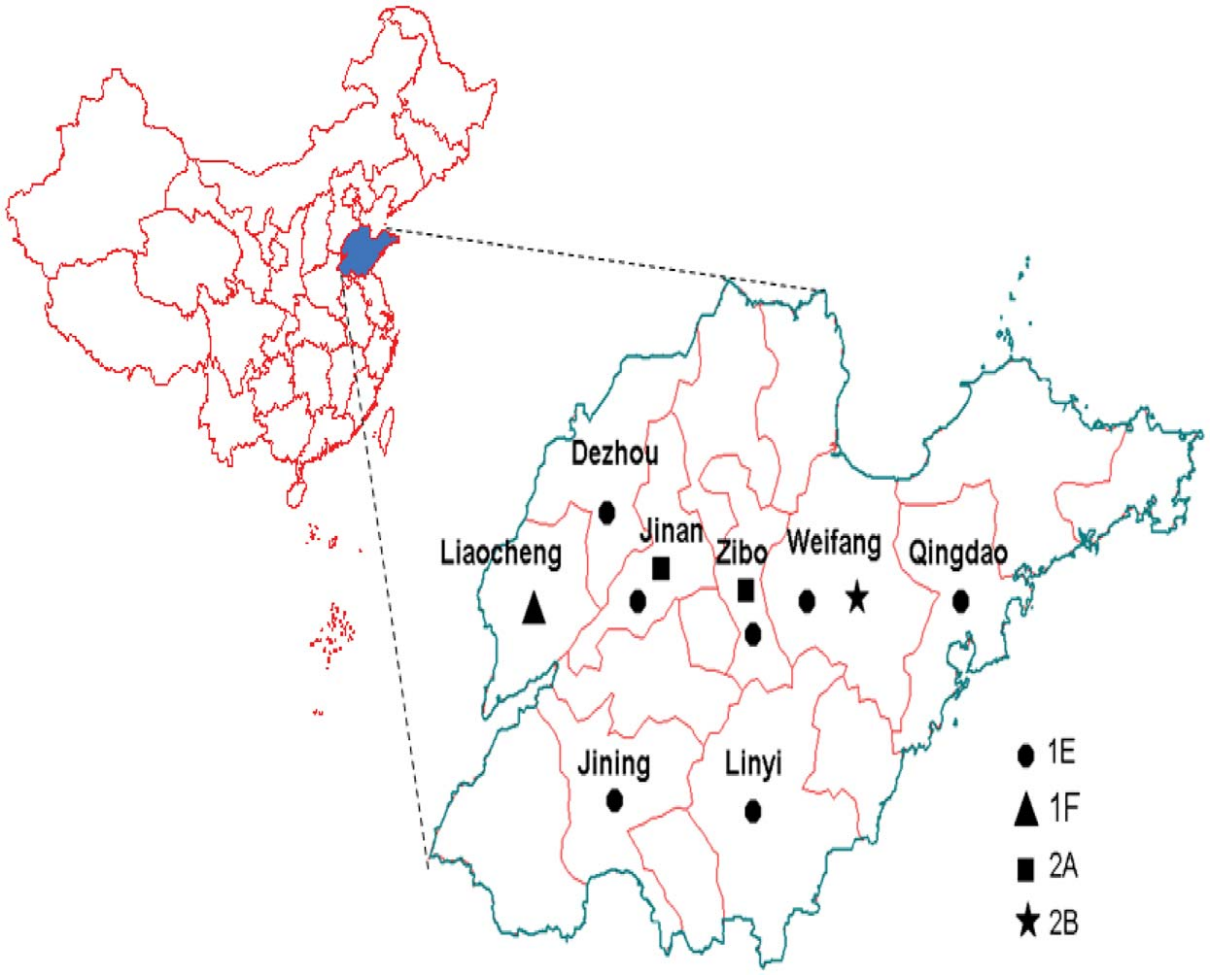
Location and genotype distribution of rubella viruses isolated in Shandong province, China during 2000–2009.

Viruses of genotype 1E were found in 7 of 8 prefectures in Shandong province during 2001–2009 (Figure 3), and the similarity of the nucleotide sequence among these viruses was 98.2–100%, while of the amino acid sequence was 99.1–100%; genotype 1F was only found in Liaocheng prefecture in 2000 (Figure 3), and compared with the genotype 1F WHO reference strain (strain TS38-CH-00, detected in Anhui province in 2000) [7], it shared 95.9% (nucleotide) and 99.1% (amino acid) identity; and 3 genotype 2A viruses from Jinan and Zibo prefectures were isolated from 3 sporadic patients (Figure 3). The nucleotide sequences of the 3 viruses were very closely related to that of the domestic vaccine strain (BRD II virus); among them, 2 were identical and the other had only a 1-nt difference compared with the vaccine strain. Although the information of the immunization histories were not available for the 3 patients, the sequence information indicated that these 3 isolates likely originated from vaccines independently, and genotype 2A is considered to be inactive; genotype 2B was only isolated in Weifang prefecture in 2008 (Figure 3), and compared with the genotype 2B WHO reference strain (strain TS34-CH-00, detected in Anhui province in 2000) [7], it shared 98.9% (nucleotide) and 100% (amino acid) identity (Figure 2a). No viruses were isolated during 2003–2005, because it was a lower rubella epidemic period (incidence rate <1/100,000), and left a surveillance gap during this period.

### Genotype 1E Rubella Viruses Constantly Circulated in Shandong Province

Genotype 1E viruses accounted for the majority (19/24, 79%) of all the Shandong viruses, indicating that genotype 1E rubella viruses were predominant. In order to determine the molecular epidemiology of the Shandong genotype 1E strains, a phylogenetic analysis was conducted with 19 Shandong strains, 77 genotype 1E rubella virus strains from 19 other provinces in mainland China that circulated during 2001–2009 [9], [10], and 23 international genotype 1E strains circulating during 1995–2010 that represented 16 countries including France [13], Bahamas [14], Canada [14], Italy [15], USA [14], Malaysia [16], Suriname [14], Germany [15], Tunisia [13], Belarus [17], Sudan [18], UK [19], Kazakhstan, Ukraine, Russia, and Japan (Table S2 and Figure 2b). All Shandong genotype 1E strains during 2001–2009 belong to cluster 1 and interdigitated with the viruses from other provinces in mainland China. The Shandong sequences did not cluster with international sequences except for some from Russia in 2006, and from Japan in 2010 and 2011 (Figure 2b). Identical and similar sequences of genotype 1E rubella viruses isolated in Shandong province were also found in all other provinces (including eastern, central, and western China); this indicates that highly similar or identical 1E rubella virus sequences circulated in the various provinces with no apparent geographic restriction.

**Figure 4:**
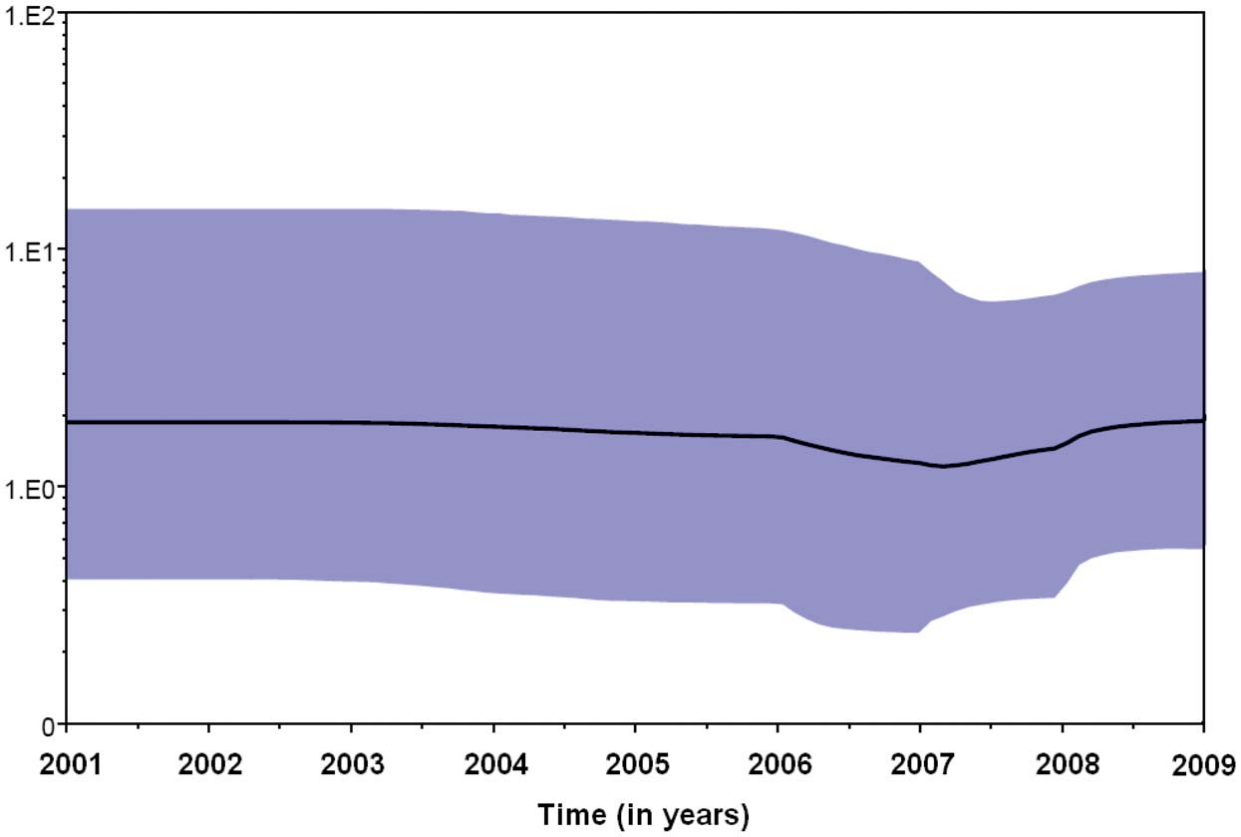
Bayesian skyline plot obtained by analyzing the 64 Beijing genotype 1E RV sequences. Ordinate: the number of effective infections at time; abscissa: time (in years). The thick solid line represents the median, and the blue area represents the 95% HPD of the number of effective infections at the time estimates.

Both the strict and relaxed clock models were implemented using the Bayesian skyline model for population growth using the Bayesian skyline plot (BSP), with the second being preferred. The BSP (Figure 4) showed that the effective number of infections in Shandong province is relatively stable from 2001 to 2009, whereas small fluctuations occurred between 2006 and 2009 even though the rubella epidemic occurred during the same period. While in the whole country, the effective number of infections remained constant during 2001–2007, when the epidemic started a decline that led to a decrease in the effective population size after 2008.

## Discussion

In Shandong province, RCV was introduced into childhood immunization schedule in 1995. Since then rubella incidence has been effectively controlled and maintained at a low level. Because there was a charge for the rubella vaccine and vaccination was voluntary before 2008, the RCV coverage was relatively lower for that time period (approximately 56–65%) (unpublished data). Therefore, the accumulation of a large number of susceptible (unvaccinated) individuals led to the occurrence of a rubella epidemic in Shandong province in 2006 and most of the rubella cases were concentrated in individuals between 15 and 19 years of age, which differed from that observed during the national rubella epidemic, this may be due to the introduction of the rubella vaccine in China since the 1990 s. The gradual shift of disease burden to the older age group has occurred in the last 12 years, and the seroprevalence of rubella antibodies in women of childbearing age in 6 prefectures of Shandong province in 2005 showed that only approximately 77.67% had antibodies against rubella [20], this is of concern because older age groups include women of childbearing age, thus raising the potential for an increase in the incidence of CRS. If high vaccine levels are not maintained, immunization of children will alter the transmission dynamics and could lead to an increase in susceptibility in young women with the potential for an increase in the incidence of congenital rubella [21]. This shift in the age at risk has already been indicated in some other countries [22]. Regions or countries attempting rubella control and elimination should ensure that routine vaccine coverage in children is greater than 85% and sustainable in the long term, and that women of childbearing age are immune [23].

WHO recommends that countries undertaking measles elimination should consider the simultaneous control and elimination of rubella [24]. Countries wishing to control and eliminate rubella should not only maintain high vaccine coverage, but also be supported by high-quality surveillance including molecular epidemiological studies [25]. Therefore, when the 3-year measles project was initiated in 1999 [12], a measles laboratory network, including 20 measles labs, was established in 17 prefectures in Shandong province, and rubella surveillance was introduced into the measles surveillance system. Virological surveillance was also set up in order to obtain important genetic baseline data.

Four genotypes including 1E, 1F, 2A, and 2B were found in Shandong province over a 12-year period after rubella vaccines were introduced. Three BRDII vaccine-associated cases were identified in Shandong province in 2001. Because vaccination campaigns were usually administrated when rubella outbreaks occurred, so isolations of vaccine virus can easily occur when outbreaks and vaccination campaigns occur simultaneously. Therefore, future isolation of the RA27/3 and BRDII vaccine-related viruses is found more frequently due to the increase doses of rubella vaccine used in China.

Genotype 2B viruses in Shandong province in 2008 had a high similarity with the virus from Anhui province in 2000 and belong to the same lineage, which indicated that genotype 2B virus circulated continuously in China for at least 8 years. National surveillance data indicated that genotype 2B viruses were frequently found in other provinces in China in 2011 (unpublished data) and had a worldwide geographic distribution [26]. Therefore, it would be interesting to collect samples from other prefectures in Shandong province to understand the prevalence situation of the genotype 2B viruses.

Although genotype 1F was only detected in Liaocheng prefecture of Shandong province in 2000, genotype 1F virus likely was the predominant virus circulating in China during 1999–2000 and had not been isolated in China since 2002. Furthermore, viruses of genotype 1F have yet to be found in other countries [9], [10].

Consistent with national reports [9], genotype 1E virus was also first detected in Shandong province in 2001. It then became the predominant genotype circulating in Shandong province. Chinese genotype 1E rubella virus isolates were grouped into 2 clusters [10]; all Shandong genotype 1E strains belong to cluster 1. All the genotype 1E rubella strains that circulated in different provinces in mainland China during 2001–2009 interdigitated with Shandong genotype 1E viruses and correlated well with each other chronologically. This suggests that genotype 1E virus in Shandong province did not evolve independently. Instead, genotype 1E strains co-evolved and co-circulated with those from other provinces. Genotype 1E/cluster1 virus may be specific to China and has already been found in neighboring countries including Russia (2006) and Japan (2010 and 2011), which may due to the high frequent international exchanges. Therefore, ongoing molecular epidemiological surveillance of circulating rubella viruses is necessary since phylogenetic analyses have become important tools in monitoring virus circulation.

Genotype 1F viruses were likely replaced by 1E viruses in connection with an epidemic in Shandong province in 2001. The manner in which this genotypic shift occurred is not clear. It is postulated that incomplete coverage of vaccine immunization would contribute to such replacement/shift of one epidemic strain by another during epidemics.

The phylogeny and times of divergence of the genotype 1E rubella virus lineages using a standard sequence window sampled at different times was inferred using a relaxed molecular clock model. The greater sensitivity of the non-parametric BSP method showed that the rubella virus population size in Shandong is relatively stable from 2001 to 2009, whereas small fluctuations occurred between 2006 and 2009. The results indicated that genotype 1E rubella viruses have constantly circulated since 2001, and are the predominant genotype in Shandong province.

In conclusion, the gradual shift of disease burden to the older age group occurred after RCV was introduced into the childhood immunization schedule in 1995 in Shandong province, and was due to the incomplete coverage of rubella vaccine immunization in Children (approximately 56–65% before 2008). If rubella vaccination rate in children is not high, it may lead to an increase in the proportion of susceptible women of childbearing age, thereby increasing the occurrence of the risk of CRS. Therefore, it is crucial to achieve and maintain a high level of vaccine immunization coverage rate among children and women of reproductive age. Four genotypes, including 1E, 1F, 2A, and 2B, were found in Shandong province during 2000–2009: genotype 2A originated from BRDII vaccine-associated cases; genotype 2B virus, detected in 2008, was found to be genetically related to the viruses isolated in Anhui province in 2000, which indicated that genotype 2B virus circulated continuously in China; genotype 1E, instead of genotype 1F, became the predominant genotype circulating in Shandong province after 2001. All Shandong genotype 1E viruses belong to genotype 1E/cluster 1; they have constantly circulated, and co-evolved and co-circulated, with those from other provinces since 2001.

## Materials and Methods

### Rubella Incidence and Vaccination Data Sources

Between 1999 and 2003, the number of all clinically diagnosed and laboratory confirmed rubella cases and annual rubella incidence rates were reported for each year through the rubella surveillance system in Shandong province. After 2004, the data were obtained directly from reports from the National Notifiable Diseases Reporting System (NNDRS). Both surveillance systems cover all the levels of Centers for Disease Control and Prevention (CDC), hospitals at the county-level and above, clinics at the township level, and other medical institutes in Shandong province.

### Clinical Samples

This study did not involve human participants or human experimentation; the only human materials used were throat swab samples collected for public health purposes from patients with clinically suspected rubella within the 7 days following rash onset. Written informed consent for the use of the clinical samples was obtained from all patients involved in this study. This study was approved by the second session of the Ethics Review Committee of the Chinese CDC.

### Viral Isolation and Primary Identification

All rubella viruses were isolated between 2000 and 2009 in 8 of 17 prefectures (Table S1), which covered the eastern, central, and western regions of Shandong province. Clinical specimens were inoculated onto monolayers of rabbit kidney (RK13) cells, African green monkey kidney (Vero) cells, and Vero/SLAM cells according to standard methods [9]. The presence of viral RNA was identified by using the reverse transcription-polymerase chain reaction (RT-PCR) method to amplify a 185-nt fragment of the E1 coding region as previously described [28].

### RT-PCR Amplification and Sequence Determination

RT-PCR was performed using the Titanium One-step RT-PCR kit (BD Bioscience, Palo Alto, CA, USA) to amplify a 1107-nt (nt 8656–9762) product containing the 739-nt WHO standard sequence window as previously described [9]. After the PCR products were purified using the QIAquick Gel Extraction Kit (Qiagen, Valencia, CA, USA), the amplicons were bi-directionally sequenced using an ABI PRISM 3100 Genetic Analyzer (Applied Biosystems, Hitachi, Japan).

### Phylogenetic Analysis

The 739-nt sequences of the rubella virus strains were aligned and phylogenetic analyses using the neighbor-joining Kimura two-parameter distance method were performed to understand the molecular epidemiology of the rubella virus isolated in Shandong province using the MEGA 5.03 program (Sudhir Kumar, Arizona State University, Tempe, AZ, USA) [29]; the reliability of the tree was estimated with 1000 bootstrap pseudoreplicates. Bootstrap values >80% were considered statistically significant for grouping. According to WHO recommendations [7], the primary criterion for valid genotype assignment is the proper grouping of the reference virus set included in the same analysis. A BSP under both strict and relaxed (uncorrelated log-normal distributed, UCLD) clock conditions was used to estimate the demographic history. BSP uses an MCMC method that allows estimates of effective population size over time with credibility intervals at every time depending on errors due to the phylogeny reconstruction and the stochastic nature of the coalescent process [27].

### Nucleotide Sequence Accession Numbers

The nucleotide sequences of 24 representative viruses of the 34 rubella strains that were isolated in this study have been deposited in the GenBank database, and are listed in Table S1. An additional 77 sequences of genotype 1E rubella viruses from China during 2001–2009 were retrieved from the GenBank database with accession numbers FJ875036–FJ875044, FJ875047–FJ875056, FJ875058–FJ875071, JF702819–JF702835, JF702841–JF702866, and JF702869.

## Supporting Information

Table S1 **Rubella viruses strains from Shandong province, 2000–2009.**
(DOC)

Table S2 **Representative genotype 1E strains from countries other than China used for phylogenetic analysis.**
(DOC)

## References

[1] Cooper LZ (1985) The history and medical consequences of rubella. Rev Infect Dis 7 S2–10. 3890105 10.1093/clinids/7.supplement_1.s2

[2] Lee JY, Bowden DS (2000) Rubella virus replication and links to teratogenicity. Clin Microbiol Rev 13: 571–587. 11023958 10.1128/cmr.13.4.571-587.2000 PMC88950

[3] Frey TK (1994) Molecular biology of rubella virus. Adv Virus Res 44: 69–160. 7817880 10.1016/S0065-3527(08)60328-0 PMC7131582

[4] WHO (2000) Preventing congenital rubella syndrome. Wkly Epidemiol Rec 75: 290–295. 11000741

[5] Reef SE, Strebel P, Dabbagh A, Gacic-Dobo M, Cochi S (2011) Progress toward control of rubella and prevention of congenital rubella syndrome–worldwide, 2009. J Infect Dis 204: S24–27. 21666168 10.1093/infdis/jir155

[6] Chen MH, Icenogle JB (2007) Molecular virology of rubella virus. In: Banatvala J, C Peckham, editors. Rubella Viruses Peckham Oxford, UK: Elsevier. 1–18.

[7] WHO (2005) Standardization of the nomenclature for genetic characteristics of wild-type rubella viruses. Wkly Epidemiol Rec 80: 126–132. 15850226

[8] WHO (2007) Update of standard nomenclature for wild-type rubella viruses, 2007. Wkly Epidemiol Rec 82: 216–222. 17571447

[9] Zhu Z, Abernathy E, Cui A, Zhang Y, Zhou S, et al (2010) Rubella virus genotypes in the People's Republic of China between 1979 and 2007: a shift in endemic viruses during the 2001 Rubella Epidemic. J Clin Microbiol 48: 1775–1781. 20351211 10.1128/JCM.02055-09 PMC2863877

[10] Zhu Z, Cui A, Wang H, Zhang Y, Liu C, et al (2012) Emergence and Continuous Evolution of Genotype 1E Rubella Viruses in China. J Clin Microbiol 50: 353–363. 22162559 10.1128/JCM.01264-11 PMC3264136

[11] Han YR, Zhao K, Gong YX, Hao SL, Wang SZ, et al (1985) Rubella vaccine in the People's Republic of China. Rev Infect Dis 7: S79. 4001739

[12] Xu AQ, Feng ZJ, Xu WB, Wang LX, Guo WS, et al (2003) Active case-based surveillance for measles in China: lessons from Shandong and Henan provinces. J Infect Dis 187: S258–263. 12721923 10.1086/368044

[13] Vauloup-Fellous C, Hubschen JM, Abernathy ES, Icenogle J, Gaidot N, et al (2010) Phylogenetic analysis of rubella viruses involved in congenital rubella infections in France between 1995 and 2009. J Clin Microbiol 48: 2530–2535. 20463161 10.1128/JCM.00181-10 PMC2897492

[14] Zheng DP, Frey TK, Icenogle J, Katow S, Abernathy ES, et al (2003) Global distribution of rubella virus genotypes. Emerg Infect Dis 9: 1523–1530. 14720390 10.3201/eid0912.030242 PMC3034328

[15] Zheng DP, Zhu H, Revello MG, Gerna G, Frey TK (2003) Phylogenetic analysis of rubella virus isolated during a period of epidemic transmission in Italy, 1991–1997. J Infect Dis 187: 1587–1597. 12721939 10.1086/374972

[16] WHO (2005) Marburg haemorrhagic fever, Angola–update. Wkly Epidemiol Rec 80: 141–142. 15898271

[17] Hubschen JM, Yermalovich M, Semeiko G, Samoilovich E, Blatun E, et al (2007) Co-circulation of multiple rubella virus strains in Belarus forming novel genetic groups within clade 1. J Gen Virol 88: 1960–1966. 17554029 10.1099/vir.0.82580-0

[18] Omer A, Abdel Rahim EH, Ali EE, Jin L (2010) Primary investigation of 31 infants with suspected congenital rubella syndrome in Sudan. Clin Microbiol Infect 16: 678–682. 19732080 10.1111/j.1469-0691.2009.02966.x

[19] Jin L, Thomas B (2007) Application of molecular and serological assays to case based investigations of rubella and congenital rubella syndrome. J Med Virol 79: 1017–1024. 17516526 10.1002/jmv.20847

[20] Song XQ, Xu A, Wang C, Xiong P, Zhang L, Xiao Z (2007) Study on Rubella Susceptibility in Pregnant Women in Shandong in 2005. Preventive Medicine Tribune 7: 577–579.

[21] Vynnycky E, Gay NJ, Cutts FT (2003) The predicted impact of private sector MMR vaccination on the burden of Congenital Rubella Syndrome. Vaccine 21: 2708–2719. 12798608 10.1016/s0264-410x(03)00229-9

[22] Castillo-Solorzano C, Carrasco P, Tambini G, Reef S, Brana M, et al (2003) New horizons in the control of rubella and prevention of congenital rubella syndrome in the Americas. J Infect Dis 187: S146–152. 12721906 10.1086/368034

[23] WHO (2011) Rubella vaccines: WHO position paper. Wkly Epidemiol Rec 86: 301–316. 21766537

[24] WHO (2000) Rubella vaccines: WHO position paper. Wkly Epidemiol Rec 75: 161–172.

[25] WHO (2006) Global distribution of measles and rubella genotypes–update. Wkly Epidemiol Rec 81: 474–479. 17175602

[26] Abernathy ES, Hubschen JM, Muller CP, Jin L, Brown D, et al (2011) Status of global virologic surveillance for rubella viruses. J Infect Dis 204: S524–532. 21666209 10.1093/infdis/jir099

[27] Drummond AJ, Rambaut A, Shapiro B, Pybus OG (2005) Bayesian coalescent inference of past population dynamics from molecular sequences. Mol Biol Evol 22: 1185–1192. 15703244 10.1093/molbev/msi103

[28] Zhu Z, Xu W, Abernathy ES, Chen MH, Zheng Q, et al (2007) Comparison of four methods using throat swabs to confirm rubella virus infection. J Clin Microbiol 45: 2847–2852. 17596370 10.1128/JCM.00289-07 PMC2045274

[29] Tamura K, Dudley J, Nei M, Kumar S (2007) MEGA4: Molecular Evolutionary Genetics Analysis (MEGA) software version 4.0. Mol Biol Evol 24: 1596–1599. 17488738 10.1093/molbev/msm092

