# Supplementary material for: Rubella Epidemics and Genotypic Distribution of the Rubella Virus in Shandong Province, China, in 1999–2010
Source: PLoS One. 2012 Jul 24;7(7):e42013. doi: 10.1371/journal.pone.0042013 (PMC3404038; doi:10.1371/journal.pone.0042013)
Supplement: Table S1 — Rubella viruses strains from Shandong province, 2000–2009. (DOC) [file pone.0042013.s001.doc]

**Table S1**. Rubella viruses strains from Shandong province, 2000–2009

| Prefecture(number of strains) | Strain | GenBank accession no. |
| --- | --- | --- |
| Liaocheng (1) ^a^ | RVi/Liaocheng.Shandong.CHN/15.00/1[1F] | AY968213^c^ |
| Jinan (4) ^a^ | RVi/Jinan.Shandong.CHN/8.06[1E] | JQ639404 |
|  | RVi/Jinan.Shandong.CHN/10.06[1E] | FJ875033 |
|  | RVi/Jinan.Shandong.CHN/13.06[1E] | JQ639405 |
|  | RVi/Jinan.Shandong.CHN/14.06[1E] | JQ639406 |
| Zibo (6) ^a^ | RVi/Zibo.Shandong.CHN/40.01/1[2A] | FJ875030 |
|  | RVi/Zibo.Shandong.CHN/40.01/2[2A] | FJ875031 |
|  | RVi/Zibo.Shandong.CHN/15.09/3[1E](4)^b^ | JF702837 |
| Jining (5) ^a^ | RVi/Jining.Shandong.CHN/40.01[2A] | FJ875032 |
|  | RVi/Jining.Shandong.CHN/20.01[1E] | FJ875029 |
|  | RVi/Jining.Shandong.CHN/14.09/1[1E](2) ^b^ | JF702840 |
|  | RVi/Jining.Shandong.CHN/14.09/3[1E] | JF702839 |
| Dezhou (3) ^a^ | RVi/Dezhou.Shandong.CHN/35.02/5[1E](3)^b^ | AY968210 ^c^ |
| Linyi (4) ^a^ | RVi/Linyi.Shandong.CHN/2.07[1E] | JQ639407 |
|  | RVi/Linyi.Shandong.CHN/5.07/1[1E] | JQ639408 |
|  | RVi/Linyi.Shandong.CHN/5.07/2[1E] | JQ639409 |
|  | RVi/Linyi.Shandong.CHN/6.07[1E] | JQ639410 |
| Qingdao (3) ^a^ | RVi/Qingdao.Shandong.CHN/13.08[1E] | JQ639411 |
|  | RVi/Qingdao.Shandong.CHN/14.08[1E] | JF702867 |
|  | RVi/Qingdao.Shandong.CHN/16.08[1E] | JF702868 |
| Weifang (8) ^a^ | RVi/Weifang.Shandong.CHN/25.08/2[2B](2)^b^ | JF702870 |
|  | RVi/Weifang.Shandong.CHN/12.09/1[1E](2)^b^ | JF702836 |
|  | RVi/Weifang.Shandong.CHN/12.09/2[1E](2)^b^ | JQ639412 |
|  | RVi/Weifang.Shandong.CHN/15.09/1[1E](2)^b^ | JF702838 |

^a^ Total number of rubella virus isolates obtained from each prefecture.

^b^ Number of identical sequences found in the same outbreak.

^c^ Rubella viruses selected as WHO reference strains in 2004.
