# Supplementary material for: Rubella Epidemics and Genotypic Distribution of the Rubella Virus in Shandong Province, China, in 1999–2010
Source: PLoS One. 2012 Jul 24;7(7):e42013. doi: 10.1371/journal.pone.0042013 (PMC3404038; doi:10.1371/journal.pone.0042013)
Supplement: Table S2 — Representative genotype 1E strains from countries other than China used for phylogenetic analysis. (DOC) [file pone.0042013.s002.doc]

**Table S2.** Representative genotype 1E strains from countries other than China used for phylogenetic analysis

| Virus isolate | Isolation country and year | GenBank accession no. | Reference |
| --- | --- | --- | --- |
| RVs/Caen.FRA/23.95/1E | France, 1995 | FN546967 | [13] |
| FRI-BAH97 | Bahamas, 1997 | AY326359 | [14] |
| DES/MB-CAN97 | Canada, 1997 | AY326358 | [14] |
| 6423/PV-ITALY-1997 | Italy, 1997 | AY161374 | [15] |
| CAS/FL-USA97 | USA, 1997 | AY326356 | [14] |
| S633-SUR98 | Suriname, 1998 | AY326363 | [14] |
| G432-GER99 | Germany, 1999 | AF551761 | [15] |
| CAB/NY-USA00 | USA, 2000 | AY326355 | [14] |
| M1-MAL-2001 | Malaysia, 2001 | AY968211 | [16] |
| RVs/Angers.FRA/36.03[1E] | France, 2003 | FN547016 | [13] |
| RVs/TUN/7.03[1E] | Tunisia, 2003 | FN547014 | [13] |
| RVi/Minsk.BLR/24.04/1[1E] | Belarus, 2004 | [AM258954](http://www.ncbi.nlm.nih.gov/entrez/viewer.fcgi?db=nuccore&val=144225253) | [17] |
| Rvi/Deweim.SDN/24.05[1E]CRS | Sudan, 2005 | FJ775000 | [18] |
| RVi/Minsk.BLR/18.05/2[1E] | Belarus, 2005 | AM258955 | [17] |
| RVs/London.GBR/08.05[1E]CRS | UK, 2005 | [EF210051](http://www.ncbi.nlm.nih.gov/entrez/viewer.fcgi?db=nuccore&id=124263498) | [19] |
| RVs/Lille.FRA/25.05[1E] | France, 2005 | FN547019 | [13] |
| Bar4-108.RUS/06 | Russia, 2006 | EF421978 |  |
| RVi/Moscow.RUS/03.06[1E] | Russia, 2006 | FJ711682 |  |
| RVi/Almaty.KAZ/13.06[1E] | Kazakhstan, 2006 | FJ711684 |  |
| RVs/Chernivtcsi.UKR/13.07[1E] | Ukraine, 2007 | FJ711683 |  |
| RVi/Vladimir.RUS/9.08[1E] | Russia, 2008 | FJ711681 |  |
| RVi/Toyama.JPN/31.10 | Japan, 2010 | AB646368 |  |
| RVs/Kawasaki.JPN/17.11/2[1E] | Japan, 2011 | AB674471 |  |
